# Neuroimaging signatures predicting motor improvement to focused ultrasound subthalamotomy in Parkinson’s disease

**DOI:** 10.1038/s41531-022-00332-9

**Published:** 2022-06-03

**Authors:** Sue-Jin Lin, Rafael Rodriguez-Rojas, Tobias R. Baumeister, Christophe Lenglos, Jose A. Pineda-Pardo, Jorge U. Máñez-Miró, Marta del Alamo, Raul Martinez-Fernandez, Jose A. Obeso, Yasser Iturria-Medina

**Affiliations:** 1 Neurology and Neurosurgery Department, Montreal Neurological Institute, McGill University, Montreal, Canada; 2 McConnell Brain Imaging Centre, Montreal Neurological Institute, McGill University, Montreal, Canada; 3 Ludmer Centre for Neuroinformatics & Mental Health, McGill University, Montreal, Canada; 4 HM CINAC (Centro Integral de Neurociencias Abarca Campal), Hospital Universitario HM Puerta del Sur, Mostoles. HM Hospitales, Madrid, Spain; 5 Network Center for Biomedical Research on Neurodegenerative Diseases, Carlos III Institute, Madrid, Spain; 6 Universidad CEU-San Pablo, Madrid, Spain

**Keywords:** Parkinson's disease, Parkinson's disease

## Abstract

Subthalamotomy using transcranial magnetic resonance-guided focused ultrasound (tcMRgFUS) is a novel and promising treatment for Parkinson’s Disease (PD). In this study, we investigate if baseline brain imaging features can be early predictors of tcMRgFUS-subthalamotomy efficacy, as well as which are the post-treatment brain changes associated with the clinical outcomes. Towards this aim, functional and structural neuroimaging and extensive clinical data from thirty-five PD patients enrolled in a double-blind tcMRgFUS-subthalamotomy clinical trial were analyzed. A multivariate cross-correlation analysis revealed that the baseline multimodal imaging data significantly explain (*P* < 0.005, FWE-corrected) the inter-individual variability in response to treatment. Most predictive features at baseline included neural fluctuations in distributed cortical regions and structural integrity in the putamen and parietal regions. Additionally, a similar multivariate analysis showed that the population variance in clinical improvements is significantly explained (*P* < 0.001, FWE-corrected) by a distributed network of concurrent functional and structural brain changes in frontotemporal, parietal, occipital, and cerebellar regions, as opposed to local changes in very specific brain regions. Overall, our findings reveal specific quantitative brain signatures highly predictive of tcMRgFUS-subthalamotomy responsiveness in PD. The unanticipated weight of a cortical-subcortical-cerebellar subnetwork in defining clinical outcome extends the current biological understanding of the mechanisms associated with clinical benefits.

## Introduction

Dopamine (DA) replacement therapy with levodopa and DA agonizts along with deep brain stimulation (DBS) are still the pillar of symptomatic treatment of Parkinson´s disease (PD)^1,2^. The latter has become customary treatment of PD patients with levodopa-induced motor complications worldwide^1,3–5^. However, the invasive nature of DBS surgery, patient’s reluctancy for wearing an implanted device and socieconomics constraints in several countries make DBS not suitable for ever^4,6^.

Transcranial magnetic resonance-guided focused ultrasound (tcMRgFUS) has been recently used to treat neurological conditions through therapeutic thermoablation of selected brain regions^7^. In PD, tcMRgFUS has been proposed as a non-invasive alternative to DBS^8,9^. Although the focused ultrasound technique has been established for years, recent technical advances with an MR-guided device allows ultrasound waves to be delivered into target brain regions more precisely, generating focal “ablations”^10,11^. The neurosurgical targets used for ablation and DBS in PD, i.e. the thalamic ventralis intermedium (Vim)^12^, the subthalamic nucleus (STN)^13^, and the globus pallidum pars interna (GPi)^14^, are also targeted with tcMRgFUS. The procedure also aims to disrupt abnormal neuronal activity in the motor circuit, thus achieving the desired anti-parkinsonian effect^4,8^. Focused ultrasound thalamotomy is accepted for the treatment of tremor-dominant PD, while unilateral subthalamotomy and pallidotomy have been mainly used in patients with asymmetric parkinsonism and/or levodopa-induced motor complications^9^.

Overall, patients have shown promising improvements with tcMRgFUS in several PD motor features. Unilateral subthalamotomy improved all parkinsonian cardinal motor signs significantly^10,15^, and thalamotomy provoked 60% improvements in tremor scores^16–20^. Furthermore, pallidotomy has been shown to reduce levodopa-induced dyskinesias while only modest improvement of parkinsonism^21^. However, variability in treatment benefits as well as the presence of side effects is a common finding in the performed studies^17,21,22^. Importantly, lesion location and morphometric measures are the main source for variability, but other patient-specific factors could contribute as well to safety and efficacy outcomes^23^. The fact that not all patients achieve the same clinical improvement after tcMRgFUS reflects the crucial need for a quantitative pretreatment individually-tailored prediction of potential effects. Furthermore, although tcMRgFUS is undeniably a clear therapeutic advance for PD^15^, limited associations between observed clinical outcomes and concurrent brain re-organization effects have been explored. This implies that the multisystem neurophysiological mechanisms of individual responsiveness to tcMRgFUS remains unclear.

**Fig. 1:**
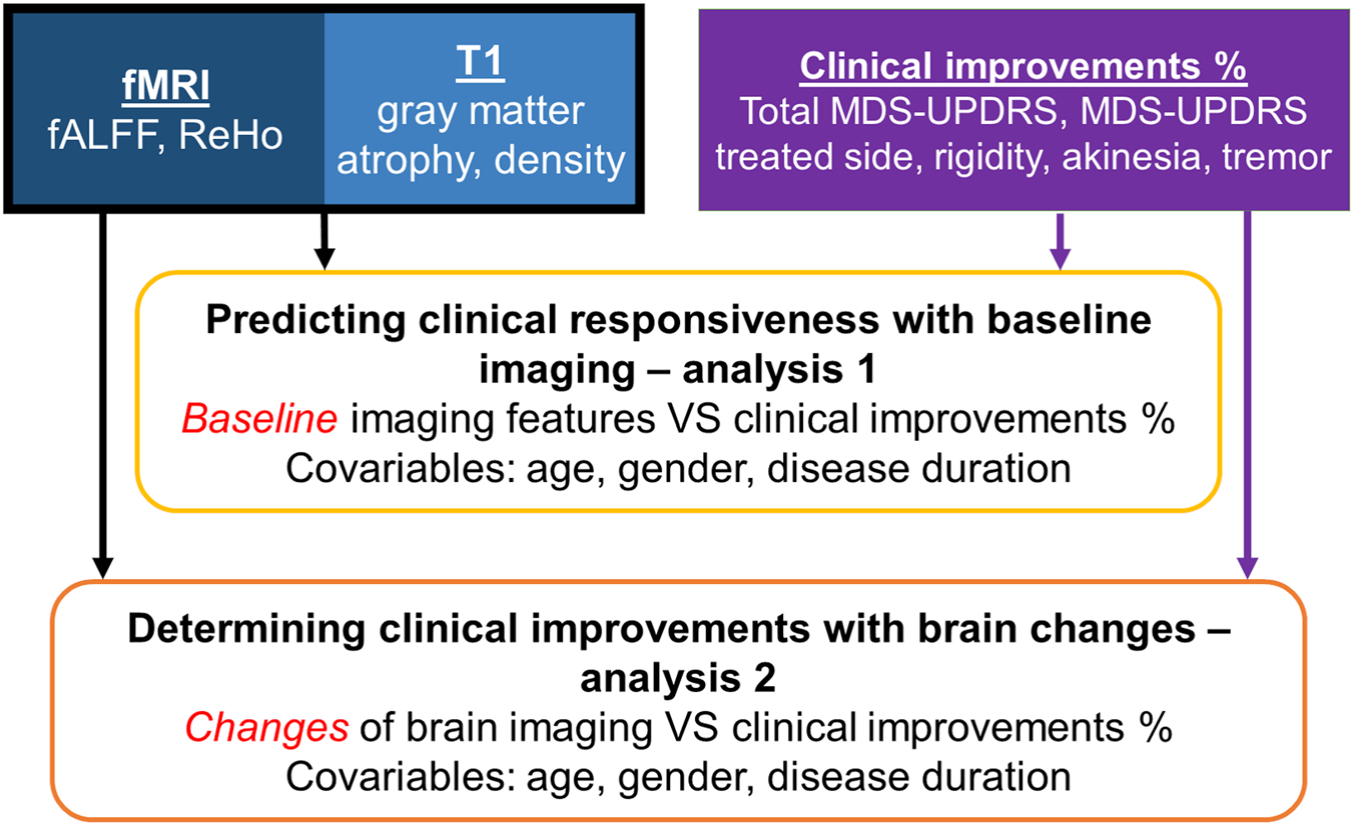
Flowchart of acquired data and two main study analyses. The purpose of two analyses and the used data for each of them are outlined in the figure.

The two main purposes of this study were to test if imaging-derived profiles before tcMRgFUS-subthalamotomy can predict clinical responsiveness and whether or not treatment-induced functional and structural brain changes can explain observed clinical outcomes (Fig. 1). To this end, we used multivariate statistical techniques to analyze MRI-based profiles in relation with treatment response.

## Results

### tcMRgFUS-subthalamotomy improves motor features remarkably

**Fig. 2:**
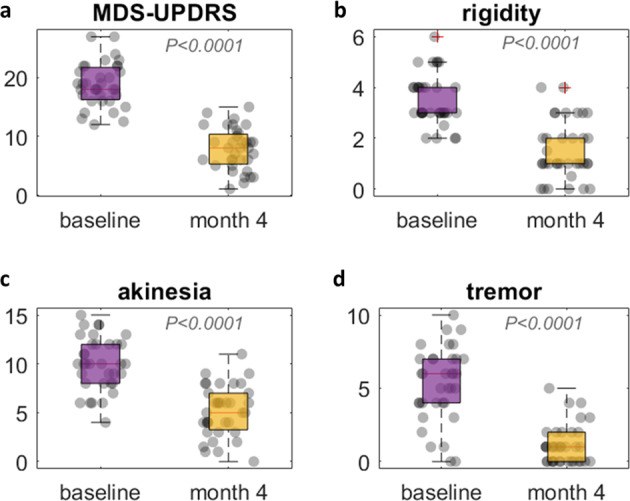
Raw clinical scores at baseline and month 4. Four clinical variables (**a**–**d**) are shown with *P*-values in paired *t*-tests, indicating significant differences before and after treatment in all motor assessments. Each dot represents a subject. Center lines indicate the median. Box limits indicate 75th and 25th percentile. Red crosses are outliers. All scores are corresponding to the treated side.

Subthalamotomy improved unilateral motor signs significantly (Fig. 2 and Table 1, notice that the more negative the differential values reported for a clinical variable, the higher the patients’ improvement on the corresponding domain in Table 1). In brief, all the clinical motor items were significantly reduced (*P* < 0.0001) after treatment in paired *t*-tests with MDS-UPDRS III total, MDS-UPDRS III treated side, rigidity, akinesia, and tremor. Overall, tremor was reduced the most with 74% change, followed by a 60% improvement for both rigidity and MDS-UPDRS III at the treated side. Akinesia and the total MDS-UPDRS III scores reduce by 47% and 40%, respectively (Table 1 and Fig. 2).

**Table 1:** Demographics and clinical assessments of 35 patients with PD. All the clinical assessments were done in off-medication state. Rigidity, akinesia, and tremor scores are corresponding to the treated side.

Demographics	Mean ± STD	
Age	56.6 ± 9.5	
Gender	22 males, 13 females	
Disease duration in years	7.2 ± 2.8	
Treated side	16 right side, 19 left side	
**Clinical assessments**	**Baseline**	**Month 4**
MDS-UPDRS III—total scores*	37.6 ± 8.0	22.9 ± 8.6
MDS-UPDRS III—treated side*	18.8 ± 3.7	7.8 ± 3.6
MDS-UPDRS III—rigidity*	3.5 ± 1.0	1.5 ± 1.2
MDS-UPDRS III—akinesia*	10.0 ± 2.7	5.2 ± 2.6
MDS-UPDRS III—tremor*	5.3 ± 2.5	1.1 ± 1.3
**Clinical changes (%)**		
MDS-UPDRS III—total scores	−40.0 ± 18.0	
MDS-UPDRS III—treated side	−57.7 ± 19.0	
MDS-UPDRS III—rigidity	−58.0 ± 30.7	
MDS-UPDRS III—akinesia	−47.1 ± 27.4	
MDS-UPDRS III—tremor	−74.0 ± 30.5	

^*^Two-sided paired *t*-test *P* < 0.0001 [MDS-UPDRS The Movement Disorder Society-sponsored Revision of the Unified Parkinson’s Disease Rating Scales].

### Baseline neuroimaging signature predicts clinical outcomes

**Fig. 3:**
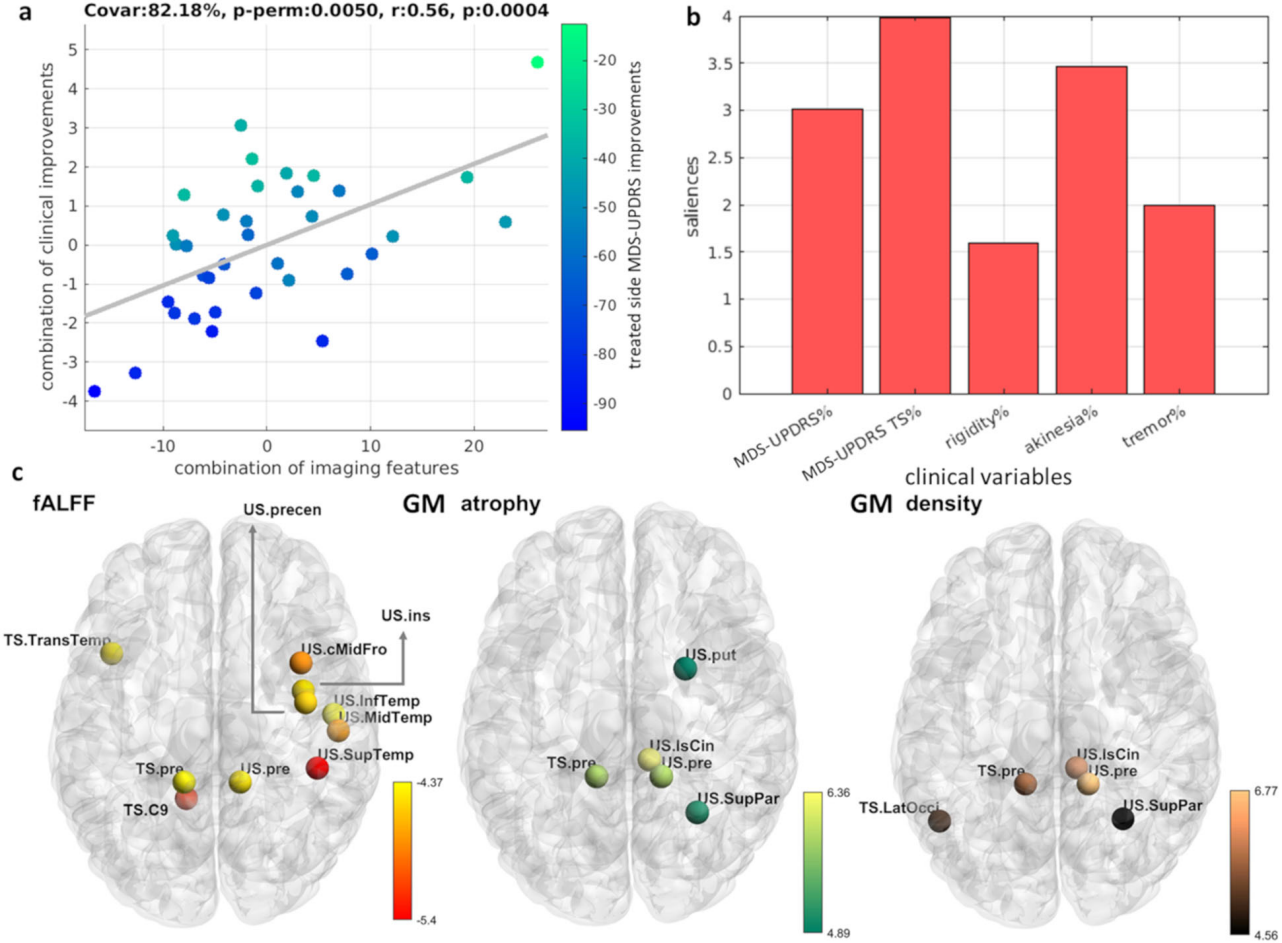
Baseline imaging association with tcMRgFUS-subthalamotomy outcomes. **a** The combinations between imaging features and clinical variables are significantly correlated. Each dot represents one subject and is color-coded with the MDS-UPDRS changes percent (specific to the treated side). **b** Contribution of clinical outcomes (month-4 visit) to the observed association with the baseline imaging. Each bar indicates the salience/importance of the variables (i.e. bootstrapping ratio). **c** The contributions of top 5% imaging regional features. Baseline fALFF, gray matter atrophy, and gray matter density contributions are shown on the left, middle, and right, respectively. Color bars indicate salience/importance (i.e. bootstrapping ratio) in each component. Of note, as atrophy values are negative by definition, bigger absolute values represent greater atrophy. [fALFF fractional amplitude of low-frequency fluctuation, US untreated side, TS treated side, GM gray matter; the full name of each region is included in Supplementary Table S1].

We identified a significant PLS-LV component explaining about 82% of the total common imaging-clinical covariance (*P* < 0.01, FWE-corrected; Fig. 3). Improvements in most clinical assessments were consistently associated with the baseline imaging data. A bootstrapping procedure confirmed that all considered clinical variables were robustly associated with the imaging data, i.e. bootstrap CIs of the clinical variables’ salience/contribution did not cross the zero value (Fig. 3b, Supplementary Fig. S1). For early imaging predictors, the most influential features (top 5% predictors from the bootstrapped PLS) included amplitude of the low frequency neural fluctuations at rest (fALFF) in the temporal cortex, and gray matter density in the putamen and posterior part of the brain (Fig. 3c; Table 2). Higher baseline fALFF values were associated with stronger treatment-induced clinical improvements (Fig. 3c). Contrary, higher structural atrophy at baseline in the putamen and posterior regions in the brain (precuneus, isthmus cingulate, occipital, and parietal gyrus) were associated with weaker clinical improvements (Fig. 3c).

**Table 2:** Mean contributions of each imaging modality in the significant latent variables space. Contribution values range from 0 to 1, indicating the fraction of total contribution among other modalities.

Threshold of salience	fMRI fALFF	fMRI ReHo	GM atrophy	GM density
**Analysis 1: baseline features VS clinical outcomes**				
Top 5%	0.61	0	0.21	0.18
**Analysis 2: brain changes between baseline and month-4 visit VS clinical outcomes**				
Top 5%	0.62	0	0	0.38

fALFF fractional amplitude of low-frequency fluctuation, ReHo regional homogeneity, GM gray matter.

### Mapping brain changes underlying clinical improvements

**Fig. 4:**
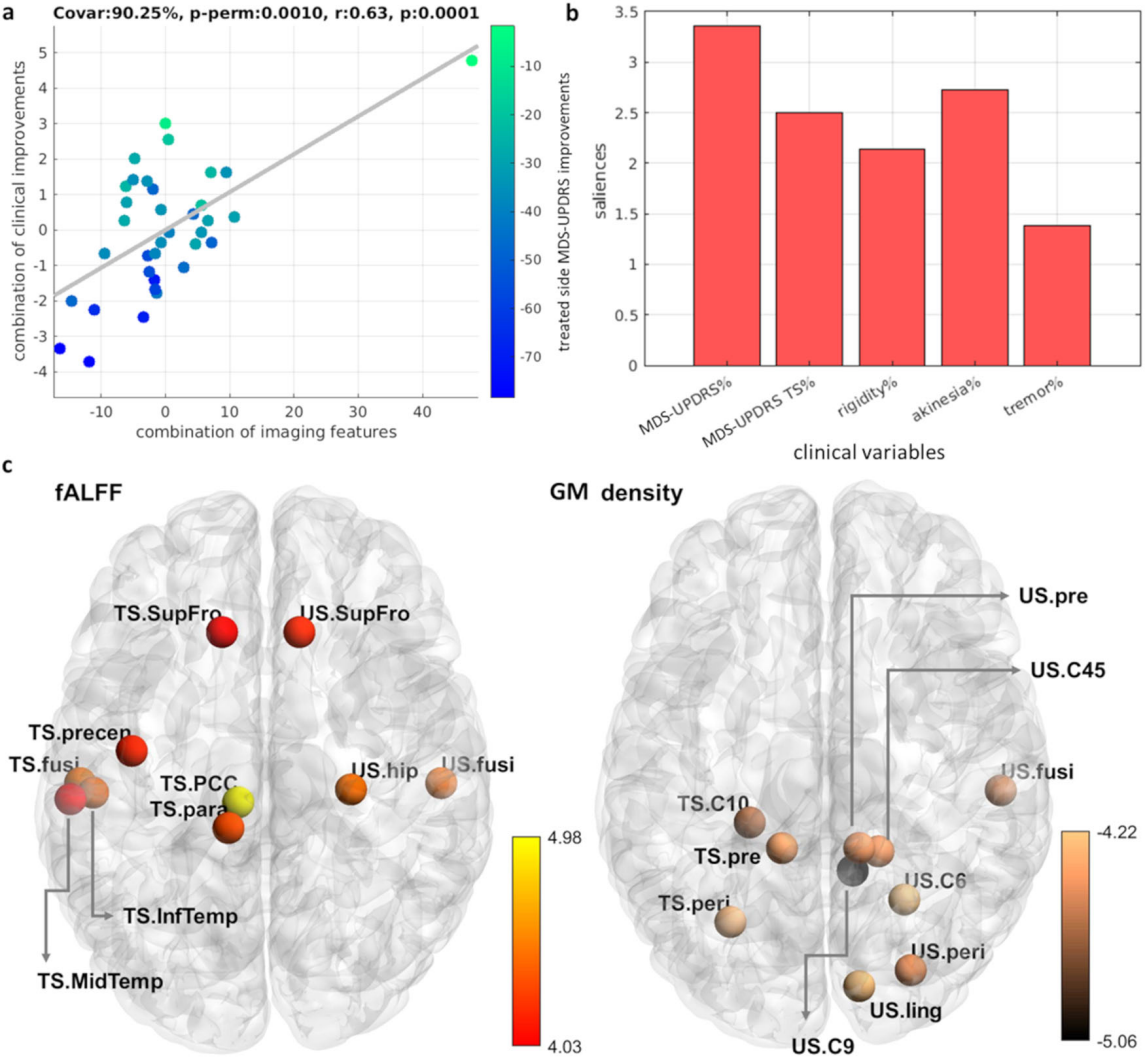
Longitudinal brain changes associated with tcMRgFUS-subthalamotomy clinical outcomes. **a** PLS-LV component explains 90.25% of the common imaging-clinical covariance. Each dot represents one subject color-coded with the total MDS-UPDRS changes in percent. **b** Contribution (i.e. bootstrapping ratio) of the five clinical outcome measures in the association with the longitudinal neuroimaging changes. **c** Contributions of the top 5% brain regional longitudinal changes after treatment. Changes in fALFF and gray matter density are shown in the left and right, respectively. [fALFF fractional amplitude of low-frequency fluctuation, TS treated side, US untreated side, GM gray matter; the full name of each region is included in Supplementary Table S1].

The analysis with brain changes and the clinical treatment outcomes revealed one significant LV component (*P* < 0.005, FWE-corrected) explaining 90% of the common imaging-clinical covariance (Fig. 4a).

Figure 4 presents the statistical contribution of the top 5% imaging features. Ten functional features and ten structural measures were significantly influential in the model (with bootstrapped 95% CIs not crossing zero; Supplementary Fig. S2). fALFF contributed the most with 62% of the total PLS-LV, followed by gray matter density with 38% (Table 2). Figure 4b, c illustrates the importance of each regional variable on the cross-correlation between datasets. All clinical improvements were positively associated with the functional changes in frontal, precentral, temporal, paracentral, and posterior cingulate regions, while were negatively correlated with gray matter differences in the posterior regions and cerebellar lobules. Thus, stronger clinical outcomes were related to structural alterations of gray matter density in the cerebellum and a few posterior cortical regions, while major patterns of functional changes regarding low frequency neural fluctuations in frontotemporal areas were associated with smaller absolute clinical outcomes (i.e. weaker improvements). Therefore, these results suggest that better treatment responsiveness is consistently associated with concurrent increasing morphometric changes of gray matter in the precuneus, cerebellum, occipital gyrus as well as decreasing amplitude of low frequency neural fluctuations among frontal/temporal/parietal areas.

## Discussion

We aimed to decode the neurobiological bases underlying the positive clinical effects of tcMRgFUS-subthalamotomy. For this, we first tested the predictability of baseline imaging to motor improvements as a step towards future patient pre-selection in clinical trials. In addition, we aimed to detect treatment-induced functional and structural brain changes underlying the observed clinical improvements. As discussed below, in addition to the considered multimodal brain signatures, other factors have the potential to impact the early prediction of treatment responses (e.g. lesion topography). Accordingly, our analysis represent an initial promising step towards the early data-driven identification of tcMRgFUS-subthalamotomy effects in PD.

We used a robust multivariate approach to explore the cross-correlation patterns between neuroimaging predictors and clinical variables^24–26^. Our results (Fig. 3) indicate that higher values of clinical improvements were associated with higher structural atrophy and fALFF values in certain regions at baseline. These findings support the existence of a specific pretreatment functional and structural brain signature predictive of individual responsiveness to tcMRgFUS-subthalamotomy. Specifically, given that both clinical improvements and atrophy values were negative, this cross-correlation pattern indicates that stronger treatment outcomes were related to higher baseline atrophy and less gray matter density in the putamen and posterior cortical regions, as well as stronger amplitude of low frequency neural fluctuations in frontal, parietal, cerebellar regions with a cluster in the temporal cortex, which may first appears as counterintuitive. A pausable explanation may be that the effects of tcMRgFUS-subthalamotomy may be more notable (in MRI and clinical evaluations) in the clinically more affected patients at baseline, with confounding factors (e.g. MRI signal to noise ratio) potentially masking subttle treatment effects in the less affected patients. We observed that patients with higher baseline MDS-UPDRS motor scores (more severe disease state) presented higher raw improvements after FUS subthalamotomy^15^. Interestingly, DBS-related findings have suggested similar mechanisms that subjects with higher baseline UPDRS scores were associated with greater improvements^13^, and trials with dopaminergic drugs in PD^27–29^ have also been associated with greater improvement in the more affected patients. Finally, as the mechanisms of impaired neural fluctuations are not fully understood, here we define level of baseline imaging impairments based on gray matter morphometry. Neuronal compensation may be a potential factor underlying the co-existence of reduced gray matter integrity and stronger amplitude of low frequency neural fluctuations, with neural information flow increasing to compensate for structural damage in order to maintain essential brain functions^30^.

Our results suggest that treatment responsiveness prediction in PD requires multiple features across subcortical, cortical, and cerebellar brain areas rather than specific regions solely. Functional features at baseline dominated the importance to predict clinical outcomes with a temporal lobe cluster and other distributed regions (Table 2, Fig. 3C). For top structural features, there were overlapping regions between density and atrophy measures in the precuneus, superior parietal gyrus, and isthmus cingulate cortex. The precuneus appeared to be influential across the three analyzed measures, supporting the multifaceted role of this cortical region in treatment responsiveness. Further, the precuneus has been identified as both structural and functional “hub” (highly connected area) in healthy subjects^31–33^. The precuneus may impact treatment effects given its hub characteristics, but its pathophysiological role in PD requires further investigation. Although there were relatively few baseline neuroimaging predictors in the subcortical and cerebellar areas (Fig. 3C), some regions in the deep brain nuclei and cerebellum were detected as influential when a looser statistical threshold was considered (Supplementary Table S2). The putamen is recoghized to play a key pathophysiological role in PD^8,34^, which is in line with the observed strong prediction power here for this region. Surprisingly, even with a looser threshold, only one significantly imaging predictor in the primary motor cortex was identified (i.e. precentral gyrus in the untreated side). PD pathology is well-known to go beyond the nigro-striatal system and the motor areas^35,36^, and our results suggest that indeed the inter-individual variability in tcMRgFUS-subthalamotomy effects may be determined by not only motor regions but diffuse networks which include non-motor brain areas as well. Perhaps, disease progression in the primary motor cortex is a “downstream” effect, implying less power to predict potential treatment outcomes compared to those “upstream” regions in the cerebellum-thalamus-cortical axis. This will be the focus of our future research.

Here, we assumed that traditional univariate statistics in clinical studies may not capture the crucial changes contributing to disease progression or treatment efficiency. Therefore, instead of reporting the significant imaging differences before and after treatment with a traditional univariate approach, we focused on whether the brain changes after treatment were associated with clinical outcomes in a multivariate fashion (Fig. 4.). The identified relevant structural reorganizations were located in the cerebellum contralateral to the treatment site and in the posterior regions (i.e. occipital and parietal lobes), while the functional changes were mostly in distributed areas (e.g. frontotemporal, paracentral, posterior cingulate cortices). The results indicated that (i) stronger clinical improvements were associated with increasing gray matter density changes in the cerebellum and posterior brain regions, and (ii) weaker improvements associated with increasing functional changes at rest in frontal, temporal, paracentral, and posterior cingulate cortices. By modulating the gray matter integrity of STN, the tcMRgFUS-subthalamotomy may have altered the cortico-striatal circuit, suppressing excitatory outputs from the STN to other deep gray matter regions and improving motor features^8^. Treatment-associated morphometric changes in several cerebellar regions (Supplementary Table S2) may be caused by the spread of treatment effects from the basal ganglia to alter certain aspects of motor features through cerebellum-basal ganglia-cortical connections^37,38^. Given that these connections are highly integrative across systems^37^, other cortical regions such as parietal and temporal-occipital areas may have also received treatment effects to alter sensorimotor properties. Similarly, previous functional neuroimaging studies have reported a modulatory effect of both subthalamotomy^11^ and STN-DBS^28^ on the activity of the cerebellar regions. Increasing evidence supports the cerebellum’s direct role in the pathophysiology of PD (for a review, see^38^), which involves cerebellum-basal ganglia anatomical connections, PD-associated pathological, structural and functional alterations, and potential compensatory effects^38^. Furthermore, a prior FDG-PET study revealed a metabolic effect in posterior parietal and occipital areas induced by MRgFUS-subthalamotomy^11^.

High fALFF values within a given region indicate strong low frequency neuronal activity compared to the full frequency spectrum^39^. Our results (Fig. 4) suggest that more changes of regional functional activity in the posterior cingulate cortex, precentral and paracentral gyrus, as well as a major frontotemporal cluster, were associated with *weaker* treatment outcomes. In other words, *better clinical outcomes could be linked to treatment-induced decreased regional low frequency activity in these regions*. This supports the notion that distributed cortical networks might be the key for ameliorating parkinsonian features rather than frontostriatal circuits only^40,41^. Many of these cortical regions have also been previously recognized as functional brain hubs, such as the posterior cingulate cortex, superior frontal gyrus, fusiform gyrus, and middle temporal gyrus^31,42^. Given that cortical hubs are often more affected than non-hub cortical regions in a variety of brain disorders including PD and other prevalent neurodegenerative diseases^43–45^, strengthening the integrity of these regions could potentially generate resistance against disease, or provide certain protections to maintain essential brain functions. All together, our results may reflect that the subthalamotomy initially altered brain circuits in the basal ganglia and the effects spred to not only the cerebellum but also to distributed cortical areas including hubs, which in turn contributed to further propagating the intervention effects. Such complex brain patterns were widespread beyond motor loops and frontostriatal circuits, suggesting that the treatment effects also fulfill the multifocal nature of PD^46^.

In both radiofrequency and tcMRgFUS-based treatments, lesion topography, such as precise location, total volume, and effective lesion tissue, is believed to be the major determinant of clinical outcomes^23,47^. In this study, however, our primary goal was to evaluate the predictive capacity of pretreatment imaging towards clinical outcomes, and consequently we did not include specific morphometric measures of lesions in the analysis. Our results suggest that, despite considering the pretreatment imaging data only (and not the lesion topology), it is still possible to significantly predict (*P* = 0.005, FWE-corrected; Fig. 3) the individual variability in clinical response to tcMRgFUS-subthalatomy. Although this is an interesting finding, its underlying neurobiological basis needs to be unraveled. Thus, based on the previous findings^28,47^ and our current results, it is reasonable to believe that baseline neuroimaging data may support lesion characteristics in predicting the clinical response to tcMRgFUS-subthalatomy in an heterogeneous PD population. This will be a central aim of coming related studies.

Although both tcMRgFUS and DBS have resulted in significant clinical improvements while targeting the same region (i.e. STN), the underlying mechanisms might be different at a macroscale level. DBS seemed to strongly modulate thalamocortical circuits, with imaging measures in the basal ganglia, primary motor cortex, and motor association regions being predictive of clinical improvements^13,28,29^. Meanwhile, the effects of tcMRgFUS-subthalamotomy are partially overlapped with that in DBS but with an emphasis on the cerebellum and cortical distribution. This may not be surprising considering the major role of the primary motor cortex in PD, but our findings also indicate a strong association between motor improvements and subcortical-cortical-cerebellar loops. Cerebellar DBS is also considered in many studies^48,49^, but the multimodal imaging outcomes in PD require further research. Therefore, here we propose that rather than directly impacting motor circuits in PD, tcMRgFUS-subthalamotomy preferably modulates the coordination between subcortical, cerebellar, and cortical hub regions to restore the balance across the brain.

The study has two major interpretative limitations and a number of metodhological issues all of which need to be taken into consideration. First, it is difficult to reconcile conceptually that greater atrophy in a number of cerebral regions is associated with better therapeutic outcome. Cortical atrophy in PD as measured by MRI is well-known to be preceeded by hypometabolism, together heralding cognitive impairment and a tortous clinical evolution^50^. These are not in principle good clinical features for a positive response to any therapy in PD. It may be that the correlation with atrophy is determining higher disease severity and greater parkinsonism, which are known to correlate with better motor improvement to functional interventions^51,52^. Second, the pathophysiological significance of slow oscillations (i.e. fALFF) in PD is not well understood. The parkinsonian state is best characterized by increased power in the beta band, which is recognized as a typical feature of the STN and other nuclei in PD^53,54^. Therefore, the relation between low frequency oscillations and typical beta bust shall be considered. Other technical aspects also require commenting and qualification. First, the study’s sample size is relatively small. As considering more than one variable increases sensitivity in multivariate analyses, our approach with multivariate cross-correlation has benefits in overcoming small sample sizes with a high amount of features as well as detecting treatment effects with longitudinal data^55^. Second, although the primary goal was to evaluate the predictive capacity of imaging rather than to determine the factors contributing to clinical outcomes, measures of lesion topography shall be included in future research. Third, imaging changes at month-4 visit showed small variances, i.e. small differences before vs after treatment (Fig. 4a). Given the relatively short period of time analyzed, further study of long-term brain and clinical changes is required to clarify tcMRgFUS-subthalamotomy effects. Fourth, we included both functional and structural MRI modalities to investigate neuronal fluctuations and gray matter morphometry. However, given that PD presents a multisystem nature^36^ involving proteomic and neurotransmitter abnormalities, molecular brain imaging (PET, SPECT) would provide essential information for a better understanding of underlying pathomolecular mechanisms. Furthermore, as the primary goal was to explore whether treatment outcomes were associated with neuroimaging features rather than studying the specific brain circuits only, the used brain parcellation is mostly based on major neuroanatomical landmarks. Regions with unique functions along specific pathways are not available in such parcellation, such as the association motor cortex. In order to develop efficient strategies for precision medicine, improving these limitations will be of crutial importance in our future work.

In conclusion, this study suggests that baseline neuroimaging is predictive of tcMRgFUS-subthalamotomy responsiveness in PD, and clinical improvements are explained by distributed, rather than localized, functional and structural brain changes.

## Methods

### Subjects

Thirty-eight subjects with markedly asymmetric PD were included in two clinical trials utilizing unilateral subthalamotomy with focused ultrasound at Centro Integral de Neurociencias, University Hospital HM Puerta del Sur, Móstoles, Madrid, Spain (Clinical Trial Registration number: NCT02912871, study duration: April, 2016 to January, 2017 and NCT03454425, study duration: February 27, 2018 to January 30, 2021)^10,15^. The study was approved by the HM Hospitales Ethics Committee for Clinical Research and all participants provided written consent forms. The detailed inclusion and exclusion criteria were described in the previous pilot study^10,15^. In short, the subjects were not suitable for DBS based on their clinical and demographical characteristics and subthalamotomy via tcMRgFUS was considered as the best option. They showed no severe dyskinesia, history of brain surgery and hemorrhage, unstable cardiac or psychiatric disease. Two patients presented complications (one showed non-motor problems and the other one presented mild treatment-induced paresis) and one subject did not complete clinical evaluations due to the COVID-19 pandemic, which resulted in a total of 35 subjects in the analysis.

### Intervention procedure

The procedure of unilateral subthalamotomy via tcMRgFUS was carried out in an ExAblate 4000 system (InSightec, Haifa, Israel), coupled to a 3 T GE scanner (Discovery 750w, GE Healthcare, Milwaukee, WI). The detailed procedure has been reported elsewhere^10,15^. Subthalamotomy was performed to treat each patient’s most affected hemibody (sixteen patients on the right side; nineteen patients on the left side). All subjects received baseline clinical assessments and image acquisition within 1 month prior to tcMRgFUS. After the procedure, anatomical images were acquired within 24 hours. Subjects underwent clinical and MRI evaluations at 4 months.

### Clinical assessment

For each subject, the MDS-UPDRS part III total, MDS-UPDRS part III on the treated side, rigidity, akinesia, and tremor scores were recorded in off-medication status at baseline as well as at the month-4 visit after treatment. Clinical improvements were calculated based on these scores with the following approach (Score_month 4_ – Score_baseline_)/Score _baseline_ × 100. Paired *t*-tests were carried out to evaluate whether clinical scores differed before VS after tcMRgFUS. Patient demographics and clinical scores are shown in Table 1.

### Image acquisition

All subjects underwent both T1-weighted images and resting-state fMRI acquisition at baseline and the month-4 visit. Within 24 hours after receiving the treatment, anatomical scans were also acquired in order to assess the topography of the subthalamotomy and perilesional edema, which were not included in the analysis of this study. Three-dimensional T1-weighted magnetization-prepared rapid acquisition gradient echo (MPRAGE) was used with the following parameters: 176 sagittal slices, TR = 2300 ms, TE = 3.34 ms, slice thickness = 1 mm, acquisition matrix = 256 × 256, and field-of-view (FOV) = 256 × 256 mm^2^. For the resting-state fMRI acquisition, each subject was instructed to remain still with eyes open fixated on a cross. Images were acquired with an echo-planar imaging (EPI) sequence with the following parameters: 450 temporal volumes, 35 slices with 4.0 mm thickness, TR = 2000 ms, TE = 30 ms, flip angle = 70˚, and matrix size = 64 × 64. Due to the time availability of the scanner, two subjects were scanned with 300 volumes, but the rest subjects followed the original protocol.

### Image analysis

T1-weighted images were first registered to the ICBM152 MNI template^56^ with FMRIB’s Linear Image Registration Tool (FLIRT; FSL, Oxford, UK)^57^ and underwent non-uniformity correction using the N3 algorithm^50^. Next, images were segmented into gray matter, white matter, and cerebrospinal fluid (CSF) probabilistic maps using SPM12 (UCL, London, UK). Gray matter segmentations were further standardized to MNI space using the DARTEL tool^58^ and each map was modulated in order to preserve the total amount of signal/tissue. Mean gray matter density and determinant of the Jacobian (DJ) values were calculated for 104 brain regions including 70 cortical regions, 16 subcortical nuclei, and 18 cerebellar areas, which constituted a robust local measure of structural atrophy in each region. These regions were determined based on the cortical Desikan–Killiany Atlas plus subcortical areas and AAL cerebellar lobules^59,60^. The measures of atrophy (from DJ) were mostly negative values, while density measures were positive values. A list of these regions is included in Supplementary Table 1.

Preprocessing steps for resting-state fMRI were carried out in FSL and SPM12, which included: (1) removing the first 10 temporal volumes to avoid unstable signals, (2) motion correction, (3) slice timing correction, (4) coregistration between fMRI and T1 image with brain masks applied, (5) spatial normalization to MNI space^56^ using the registration parameters obtained for the structural T1 image, and (6) signal filtering to keep only low-frequency fluctuations (0.01–0.08 Hz). Finally, fMRI signals were linearly detrended and motion parameters were regressed out. In order to have regional quantitative indicators of the brain’s functional integrity, the fractional amplitude of low-frequency fluctuation (fALFF)^39^ and regional homogeneity (ReHo)^61^ were calculated for each brain region mentioned in the analysis of the gray matter. All functional measures were positive values.

Nodal measures for subjects who received tcMRgFUS on the right side were “flipped” to the left side. As a result, the final imaging features for all subjects were labeled as treated and untreated side.

### Multivariate analysis

Multidimensional associations between individual neuroimaging profiles and tcMRgFUS-subthalamotomy’s clinical outcomes were tested via two multivariate cross-correlation analyses (Fig. 1). Specifically, we used partial least square (PLS) cross-correlation, which employs a joint singular value decomposition (SVD) on the covariance matrix of two different datasets^24–26^. This approach seeks the linear combinations of latent variables (LVs), within two sets of data (imaging, clinical), that maximally covariate with each other.

Our analysis consisted of two independent experiments (Fig. 1). Motivated by the fact that early individually tailored prediction of treatment efficiency remains traditionally unexplored in PD^62^, we proceed to test whether neuroimaging-derived structural and functional brain patterns at baseline could predict tcMRgFUS-subthalamotomy clinical outcomes. First, all brain imaging features at *baseline* were included as predictors of treatment clinical outcomes (Fig. 1 middle). Data included regional fALFF and ReHo values characterizing functional brain activity at rest, and gray matter atrophy and density for structural properties. For clinical outcome, improvements in the total MDS-UPDRS III, treated side MDS-UPDRS III, rigidity, akinesia, and tremor unilateral scores were included. All variables (imaging and clinical) were standardized to have zero mean and standard deviation one. Age, gender, and disease duration in years were included as co-variables and regressed out in the PLS cross-correlation.

Furthermore, randomizing permutations and bootstrapping were executed (1000 iterations each) to determine the statistical significance of each PLS-LV (indicated as FWE-corrected *P*-value) and the relative salience/importance of each original variable, respectively^25^. For each data feature, the bootstrapping ratio was calculated as its original model weight divided by its standard error across the bootstrapping iterations. Confidence intervals (CI) were also examined to ensure the robustness of salience/importance from each variable. Finally, in order to quantify which modalities were contributing the most to the imaging-clinical covariance, the mean contribution of each modality, which was normalized by the number of influential features within the modality, was calculated.

In the second analysis (Fig. 1 bottom), we analyzed the multivariate relationship between the treatment-induced *changes* in all imaging features (changes from baseline to month-4) and the clinical outcomes. For each brain region and imaging feature, the treatment-induced changes were calculated as the difference between the individual values at month-4 and at baseline (i.e. feature_month 4_ – feature_baseline_), standardizing them across all subjects to have zero mean and standard deviation one. For clinical variables, the same treatment outcomes and covariables were used and all the parameters remained the same. Similarly that for the first analysis, randomizing permutations and bootstrapping were executed (1000 iterations each) to determine the statistical significance of each PLS-LV and the relative salience/importance of each original variable on the obtained multivariate patterns, respectively^25^. All the analysis were performed with in-house Matlab codes and the imaging features were visualized with BrainNet^63^.

## Supplementary information

Supplementary Materials

## Data Availability

Due to confidentiality in clinical trials, the data needs to be requested. Requests shall be addressed to R.R.-R. & Y.I.M..

## Code availability

Computational code used for statistical analysis is available on the NeuroPM lab website (for further details, see https://www.neuropm-lab.com).
