# Supplementary material for: Neuroimaging signatures predicting motor improvement to focused ultrasound subthalamotomy in Parkinson’s disease
Source: NPJ Parkinsons Dis. 2022 Jun 3;8:70. doi: 10.1038/s41531-022-00332-9 (PMC9166695; doi:10.1038/s41531-022-00332-9)
Supplement: Supplementary file 1 — Supplementary Materials [file 41531_2022_332_MOESM1_ESM.pdf]

Supplementary Materials

For **Neuroimaging Signatures Predicting Motor Improvement to Focused Ultrasound Subthalamotomy in Parkinson's Disease**

Sue-Jin Lin, PhD[1,2,3], Rafael Rodriguez-Rojas, PhD[4,5]*, Tobias R. Baumeister, PhD[1,2,3], Christophe Lenglos, PhD[1,2,3], Jose A. Pineda-Pardo, PhD[4,5,6], Jorge U. Máñez-Miró, MD[4], Marta del Alamo, MD[4], Raul Martinez-Fernandez, MD, PhD[4,5], Jose A. Obeso, MD, PhD[4,5,6], Yasser Iturria-Medina PhD[1,2,3]*

[1] Neurology and Neurosurgery Department, Montreal Neurological Institute, McGill University, Montreal, Canada

[2] McConnell Brain Imaging Centre, Montreal Neurological Institute, McGill University, Montreal, Canada

[3] Ludmer Centre for Neuroinformatics & Mental Health, McGill University, Montreal, Canada

[4] HM CINAC (Centro Integral de Neurociencias Abarca Campal), Hospital Universitario HM Puerta del Sur, Mostoles. HM Hospitales, Madrid, Spain.

[5] Network Center for Biomedical Research on Neurodegenerative Diseases, Carlos III Institute, Madrid, Spain.

[6] Universidad CEU-San Pablo, Madrid, Spain.

* Correspondence to: R R-R, 70 Carlos V Avenue, Centro Integral de Neurociencias AC, Hospital Universitario HM Puerta del Sur, Mostoles 28938, Spain. Email: rrodriguez.hmcinac@hmhospitales.com; Y I-M, 3801 University Street, room NW312, Montreal Neurological Institute, McGill University, Montreal, Canada H3A 2B4. Email: yasser.iturriamedina@mcgill.ca

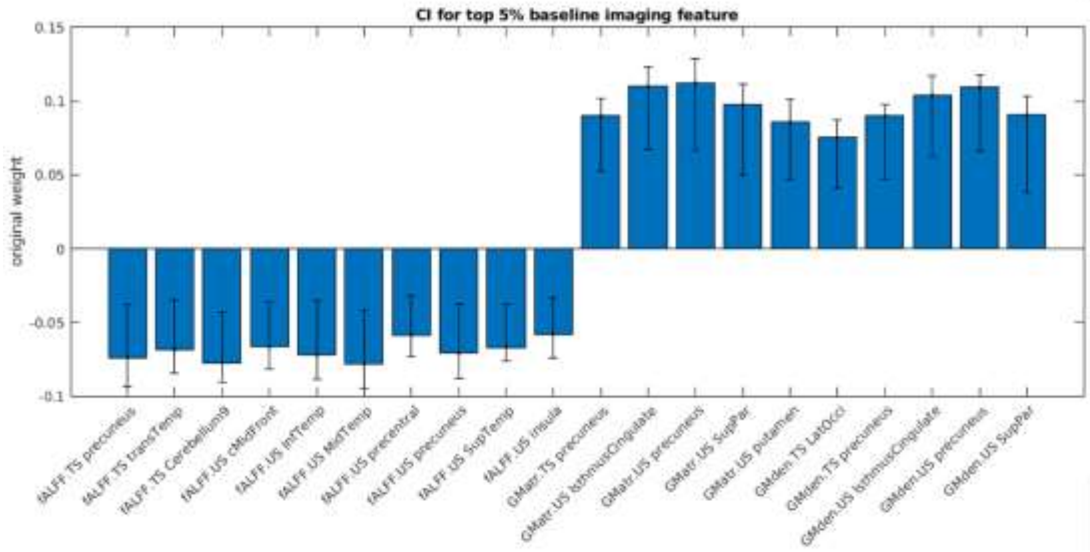

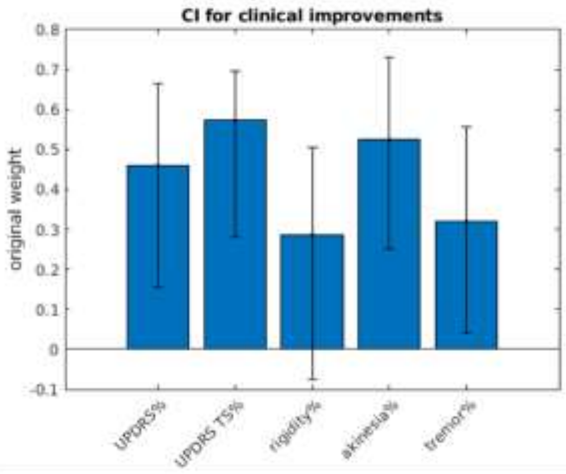

Fig. S1. Confidence intervals (CI) with 95% level of reported features in Figure 3. The left panel describes the original PLS weighting with CI of top 5% nodal features at baseline. The right panel shows the original weighting with CI of clinical improvements. Only the improvement of rigidity, which shows the smallest weight, has the CI crossing zero, indicating a less robust effect. The full label of each region is included in Supplementary Table 1. [fALFF =fractional amplitude of low-frequency fluctuation, GMatr = grey matter atrophy, GMden = grey matter density, TS = treated side, US = untreated side]

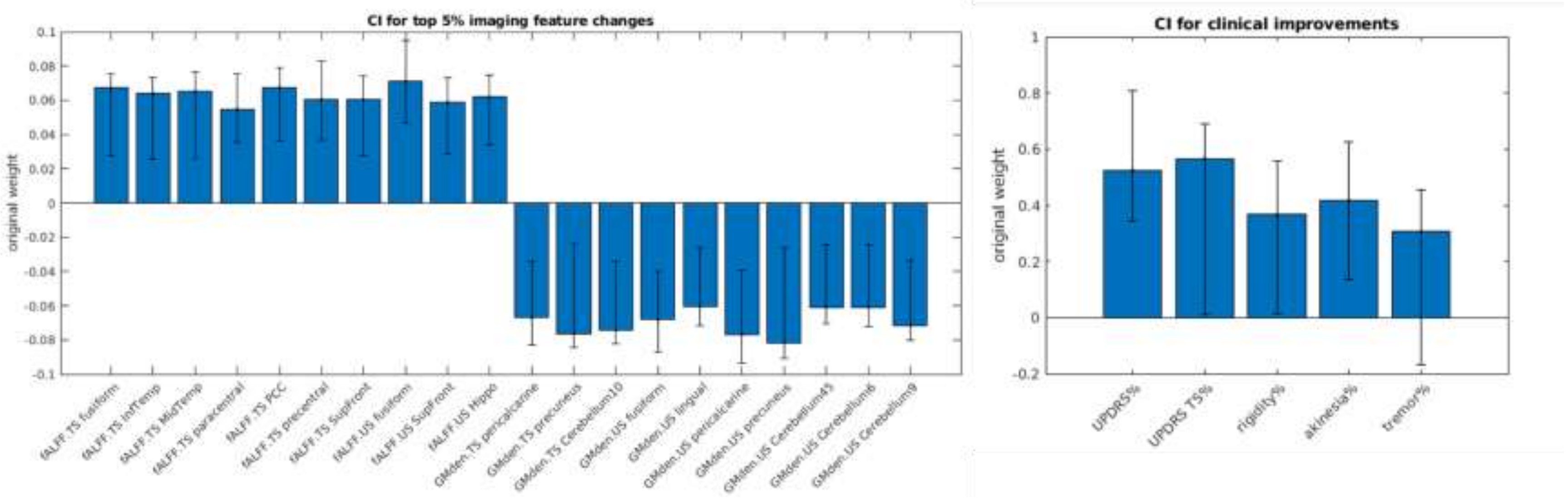

Fig. S2. Confidence intervals (CI) with 95% level of reported features in Figure 4. The left panel describes the original PLS weighting with CI of top 5% nodal feature changes. The right panel shows the original weighting with CI of clinical improvements. Only the improvement of tremor, which shows the smallest weight, has the CI crossing zero, indicating a less robust effect. The full label of each region is included in Supplementary Table 1. [fALFF =fractional amplitude of low-frequency fluctuation, GMden = grey matter density, TS = treated side, US = untreated side]

Table S1. A full list of Region-of-Interest (ROI) parcellation used in the study

**Bilateral regions – full anatomical label**	**Abbreviation in supplementary materials**	**Abbreviation in main figures**
caudal anterior cingulate cortex	cACC	cACC
caudal middle frontal gyrus	cMidFront	cMidFro
cuneus cortex	cuneus	Cuneus
entorhinal cortex	entorhinal	Ent
fusiform gyrus	fusiform	fusi
inferior parietal gyrus	InfPar	InfPar
inferior temporal gyrus	InfTemp	InfTemp
Isthmus of cingulate cortex	IsthmusCingulate	IsCin
lateral occipital cortex	LatOcci	LatOcci
lateral orbitofrontal cortex	LatOFC	LatOFC
lingual gyrus	lingual	ling
medial orbitofrontal cortex	MedOFC	MedOFC
middle temporal gyrus	MidTemp	MidTemp
parahippocampal regions	ParaHipp	Parahip
paracentral gyrus	paracentral	Para
inferior frontal gyrus - pars opercularis	pars-opercularis	p-ope
inferior frontal gyrus - pars orbitalis	pars-orbitalis	p-orb
inferior frontal gyrus - pars trianglaris	pars-triangularis	p-tri
pericalcarine cortex	pericalcarine	peri
postcentral gyrus	postcentral	postcen
posterior cingulate cortex	PCC	PCC
precentral gyrus	precentral	precen
precuneus region	precuneus	pre
rostral anterior cingulate cortex	rACC	rACC
rostral middle frontal cortex	rMidFront	rMidFro
superior frontal gyrus	SupFront	SupFro
superior parietal cortex	SupPar	SupPar
superior temporal gyrus	SupTemp	SupTemp
supramarginal gyrus	supramarginal	supra
transverse temporal gyrus	transTemp	TransTemp
insula	insula	ins
nucleus accumbens	accumbens	NAc
amygdala	amygdala	amy
basal-forebrain region	basal-forebrain	bf
caudate nucleus	caudate	cau
hippocampus	Hippo	hip
globus pallidus	pallidum	palli

putamen	putamen	put
thalamus	thalamus	tha
subthalamic nucleus	STN	STN
red nucleus	RedNucleus	RN
dentate gyrus	Dentate	den
substantia nigra	Nigra	Nigra
cerebellum crus 1	CerebellumCrus1	C1
cerebellum crus 2	CerebellumCrus2	C2
cerebellum lobule 3	Cerebellum3	C3
cerebellum lobule 4 & 5	Cerebellum45	C45
cerebellum lobule 6	Cerebellum6	C6
cerebellum lobule 7 II	Cerebellum7b	C7
cerebellum lobule 8	Cerebellum8	C8
cerebellum lobule 9	Cerebellum9	C9
cerebellum lobule 10	Cerebellum10	C10

Table S2. Top 20% influential imaging features in the multivariate cross-correlation analysis

Analysis 1		Analysis 2	
**baseline image predictors**	**importance**	**changes of imaging features**	**importance**
fALFF.TS cACC	-3.75	fALFF.TS cACC	3.71
fALFF.TS IsthmusCingulate	-3.61	fALFF.TS cMidFront	3.21
fALFF.TS paracentral	-3.27	fALFF.TS fusiform	4.43
fALFF.TS PCC	-3.87	fALFF.TS InfPar	3.32
fALFF.TS precuneus	-4.41	fALFF.TS InfTemp	4.38
fALFF.TS transTemp	-4.46	fALFF.TS IsthmusCingulate	3.57
fALFF.TS accumbens	-3.88	fALFF.TS MidTemp	4.03
fALFF.TS basal-forebrain	-3.91	fALFF.TS paracentral	4.32
fALFF.TS RedNucleus	-4.05	fALFF.TS postcentral	3.75
fALFF.TS Nigra	-3.79	fALFF.TS PCC	4.98
fALFF.TS Cerebellum9	-5.23	fALFF.TS precentral	4.16
fALFF.US cMidFront	-4.85	fALFF.TS precuneus	3.36
fALFF.US fusiform	-4.09	fALFF.TS rMidFront	3.39
fALFF.US InfTemp	-4.37	fALFF.TS SupFront	4.04
fALFF.US IsthmusCingulate	-3.34	fALFF.TS SupTemp	3.43
fALFF.US lingual	-3.80	fALFF.TS insula	3.92
fALFF.US MidTemp	-4.80	fALFF.TS Hippo	3.43
fALFF.US ParaHipp	-3.47	fALFF.TS putamen	3.75
fALFF.US paracentral	-3.31	fALFF.TS Nigra	3.97
fALFF.US PCC	-4.07	fALFF.TS CerebellumCrus1	3.40
fALFF.US precentral	-4.55	fALFF.TS CerebellumCrus2	3.34
fALFF.US precuneus	-4.47	fALFF.TS Cerebellum6	3.44
fALFF.US SupFront	-3.86	fALFF.TS Cerebellum7b	3.15
fALFF.US SupTemp	-5.40	fALFF.TS Cerebellum8	3.30
fALFF.US insula	-4.49	fALFF.TS Cerebellum9	3.29
fALFF.US amygdala	-3.27	fALFF.US cMidFront	3.58
fALFF.US basal-forebrain	-3.33	fALFF.US fusiform	4.41
fALFF.US Hippo	-4.31	fALFF.US InfTemp	3.71
fALFF.US pallidum	-3.34	fALFF.US lingual	3.21
fALFF.US thalamus	-3.42	fALFF.US MidTemp	3.90
fALFF.US Cerebellum6	-4.36	fALFF.US PCC	3.99
fALFF.US Cerebellum9	-4.33	fALFF.US precentral	3.39
ReHo.TS precuneus	-3.35	fALFF.US rMidFront	3.81
ReHo.TS SupFront	-3.34	fALFF.US SupFront	4.17
ReHo.TS accumbens	-3.23	fALFF.US caudate	3.66
ReHo.US cMidFront	-3.64	fALFF.US Hippo	4.44
ReHo.US InfTemp	-3.24	fALFF.US putamen	3.46
ReHo.US PCC	-3.67	fALFF.US Cerebellum6	3.23
GMatr.TS cuneus	3.68	ReHo.TS LatOFC	3.21
GMatr.TS fusiform	3.64	ReHo.TS SupFront	3.66
GMatr.TS LatOcci	4.10	ReHo.US PCC	3.20
GMatr.TS MedOFC	3.18	ReHo.US SupFront	3.29
GMatr.TS pars-triangularis	3.53	ReHo.US caudate	3.50
GMatr.TS precuneus	5.87	GMatr.TS cuneus	-3.55
GMatr.TS rACC	3.19	GMatr.TS pars-orbitalis	-3.14

GMatr.TS rMidFront	3.93	GMatr.TS pericalcarine	-3.78
GMatr.TS SupFront	3.18	GMatr.TS precuneus	-3.24
GMatr.TS SupPar	4.04	GMatr.TS rMidFront	-3.35
GMatr.TS accumbens	3.84	GMatr.TS CerebellumCrus2	-3.59
GMatr.TS putamen	3.56	GMatr.TS Cerebellum8	-3.16
GMatr.TS Cerebellum6	3.26	GMatr.US fusiform	-3.85
GMatr.US fusiform	3.21	GMatr.US precuneus	-3.29
GMatr.US IsthmusCingulate	6.36	GMatr.US Dentate	-3.38
GMatr.US lingual	4.14	GMatr.US CerebellumCrus1	-3.47
GMatr.US paracentral	3.17	GMatr.US CerebellumCrus2	-3.50
GMatr.US pericalcarine	4.06	GMatr.US Cerebellum6	-3.94
GMatr.US precuneus	5.90	GMatr.US Cerebellum8	-3.22
GMatr.US SupPar	5.03	GMatr.US Cerebellum9	-3.44
GMatr.US supramarginal	3.28	GMatr.US Cerebellum10	-3.38
GMatr.US amygdala	3.29	GMden.TS cuneus	-3.65
GMatr.US putamen	4.89	GMden.TS IsthmusCingulate	-3.37
GMatr.US CerebellumCrus1	3.18	GMden.TS lingual	-3.65
GMatr.US Cerebellum6	4.21	GMden.TS pericalcarine	-4.29
GMden.TS cuneus	3.36	GMden.TS precuneus	-4.35
GMden.TS IsthmusCingulate	3.29	GMden.TS rMidFront	-3.75
GMden.TS LatOcci	5.09	GMden.TS SupPar	-3.87
GMden.TS pars-triangularis	3.31	GMden.TS Cerebellum45	-3.17
GMden.TS precuneus	5.75	GMden.TS Cerebellum9	-3.87
GMden.TS rMidFront	3.17	GMden.TS Cerebellum10	-4.53
GMden.US cuneus	3.36	GMden.US fusiform	-4.51
GMden.US fusiform	3.60	GMden.US InfPar	-3.33
GMden.US InfPar	3.40	GMden.US IsthmusCingulate	-3.22
GMden.US IsthmusCingulate	6.12	GMden.US lingual	-4.23
GMden.US LatOcci	3.38	GMden.US pericalcarine	-4.40
GMden.US lingual	3.16	GMden.US precuneus	-4.38
GMden.US paracentral	3.18	GMden.US SupPar	-3.90
GMden.US precuneus	6.77	GMden.US CerebellumCrus1	-3.72
GMden.US SupPar	4.56	GMden.US Cerebellum45	-4.39
GMden.US putamen	4.23	GMden.US Cerebellum6	-4.22
GMden.US CerebellumCrus1	3.47	GMden.US Cerebellum8	-3.16
GMden.US Cerebellum45	4.22	GMden.US Cerebellum9	-5.06
GMden.US Cerebellum6	3.42	GMden.US Cerebellum10	-3.94

Note: importance is the ratio of original weights VS standard errors of the bootstrapping iterations
[TS: treated side; US: untreated side; fALFF: fractional amplitude of low-frequency fluctuation; ReHo: regional homogeneity; GMatr: grey matter atrophy; GMden: grey matter density]
